# Treatment of Fractures

**Published:** 1849-03

**Authors:** Z. Paul

**Affiliations:** Honeyove


ART. III.—*Treatment of Fractures. Cases.* By Z. PAUL, M. D. [Communicated in a letter to Dr. HAMILTON.]

Having become convinced that the practice of dressing fractures (and more especially fractures of the fore-arm) with the roller as laid down in the books, is useless, if not absolutely injurious. I have adopted a modification of the usual mode of dressing these bones, and with what success the following cases will show:—

*Case* I. Dec. 25th 1842.—Mrs. S.; aged 70. Transverse fracture of the lower third of the radius and ulna; fragments displaced.

I dressed it in the following manner. Having reduced the fracture, I placed a compress on the palmar and dorsal sides of the fore-arm, and over these a couple of splints, extending from the ends of the fingers to near the elbow, and tied them on with common tape. Leaving the limb thus lightly covered, there was very little swelling and no pain after the first dressing; the fracture united readily and without deformity.

*Case* 2. July 26th, 1846.—S. D.; a girl, aged 12. Oblique fracture of the middle portion of the fore-arm; radius and ulna both broken. Treated as the first, with the same results.

*Case* 3. July 28th, 1846.—P. R.; a boy, aged 6. Fracture of the upper third of the radius. Treated as the first; result, the same.

*Case* 4. August 30th, 1846. W. H.; a girl, aged 2. Transverse fracture of the middle portion of fore-arm; both bones broken. Treated as before: result the same.

*Case* 5. Oct. 20th, 1846.—W. B.; a boy, aged 8. Compound fracture of the middle portion of fore-arm: the radius protruded through the skin. Treated as before, with the same result.

*Case* 6. C. P.; a girl, aged 8. Oblique fracture of the lower third of fore-arm, with dislocation of the distal end of the ulna. Treatment and result the same.

In no one of the above cases was there but very little swelling, and no discoloration of the skin; and in no one was there any pain experienced by the patient.

You may ask, what is gained? I reply, nothing is lost: and I have dispensed with unnecessary bandages. But actually I think I have gained something, since I have not, under this treatment, witnessed so much swelling and inflammation.

What is required in all cases of fracture is just dressing enough to keep the bones in place; a roller will not do this without splints, and if the splints are rightly adjusted with compress, and tapes in three, or at most, four places, (i. e.) at the upper and lower ends, and one or two intermediate, they will be sufficient.

The roller keeps the limb too warm, and thus increases the inflammation, the very thing which we wish most to avoid. The swelling makes the roller and splints press unequally, and deformity is the result. In one instance I have seen inflammation, suppuration and sloughing in consequence of excessive bandaging of the arm after a fracture.

Yours truly,

Honeyoye, Dec., 1848. Z. PAUL.

If the reader will refer to the Report of the Surgical Dispensary of Buffalo Medical College, vol. 3d, page 731, of this Journal, a case will be found of fractured ulna (case No. 4) dressed in the manner detailed in the above article, and for the reasons (as stated in the report) assigned by Dr. Paul.

Editor.

